# PAP-HPV Co-Testing in Anal Cancer Screening: An Italian Experience

**DOI:** 10.3390/jcm14072186

**Published:** 2025-03-23

**Authors:** Luigi Pisano, Claudia Giachini, Martina Turco, Jacopo Farini, Filippo Caminati, Iacopo Giani, Claudio Elbetti, Simonetta Bisanzi, Stefania Cannistrà, Giampaolo Pompeo, Cristina Sani, Nicola Pimpinelli

**Affiliations:** 1 Section of Dermatology, Health Sciences Department, University of Florence, 50121 Florence, Italy; luigi.pisano88@yahoo.it (L.P.); jacopo.farini@unifi.it (J.F.); nicola.pimpinelli@unifi.it (N.P.); 2 Regional Laboratory of Cancer Prevention, Institute for Cancer Research, Prevention and Clinical Network (ISPRO), 50121 Florence, Italy; c.giachini@ispro.toscana.it (C.G.); s.bisanzi@ispro.toscana.it (S.B.); s.cannistra@ispro.toscana.it (S.C.); g.pompeo@ispro.toscana.it (G.P.); c.sani@ispro.toscana.it (C.S.); 3 SOSD Proctologia, USL Toscana Centro, 50121 Florence, Italy; filippo.caminati@uslcentro.toscana.it (F.C.); iacopo.giani@uslcentro.toscana.it (I.G.); claudio.elbetti@uslcentro.toscana.it (C.E.)

**Keywords:** anal cancer screening, PAP-HPV co-testing, high-risk human papillomavirus (HR-HPV)

## Abstract

**Background/Objectives**: Squamous cell carcinoma of the anus (SCCA) remains a relatively rare form of cancer linked to high-risk human papillomavirus (HR-HPV) infection; however, its incidence has been increasing globally. Anal cytology and HR-HPV testing can identify precursors, though standardized screening guidelines are still lacking. This study aimed to assess the correlation between high-resolution anoscopy (HRA) findings and primary screening results through PAP-HPV co-testing in high-risk patients. **Methods**: A retrospective, single-center study was conducted collecting data from the joint multidisciplinary anal cancer clinic of Piero Palagi Hospital in Florence (Italy), between August 2019 and September 2022. The sensitivity, specificity, positive predictive value (PPV), negative predictive value (NPV), and accuracy of anal cytology, HR-HPV testing, and PAP-HPV co-testing were assessed. **Results**: In 577 HRAs, histology revealed 31 AIN2+ lesions (5.4%) and 220 AIN1 lesions (38.1%), while 326 (56.5%) were negative. Cytology alone showed a sensitivity of 74.2% and specificity of 63.3% for AIN2+ lesions, while HR-HPV testing alone had a sensitivity of 96.8% and specificity of 38.1%. Co-testing demonstrated 100% sensitivity and a 100% NPV for AIN2+ lesions. Among men who have sex with men (MSM), no significant differences in outcomes were observed between HIV-positive and HIV-negative patients, likely reflecting similar high-risk behaviors and effective HIV treatments. **Conclusions**: Co-testing with anal cytology and HR-HPV testing provides the most reliable screening for high-grade lesions (AIN2+), surpassing the reliability of individual methods. Tailored co-testing strategies are crucial for early detection and effective prevention in high-risk groups.

## 1. Introduction

Currently, squamous cell carcinoma of the anus (SCCA) remains a relatively rare form of cancer, though its incidence has been increasing globally. The worldwide age-standardized incidence rate is estimated to be around 1.8 per 100,000 people per year, with variations depending on the geographic location and population demographics [1].

In the United States, approximately 8300 new cases of anal cancer, predominantly SCCA, are reported annually [2].

MSM living with HIV are the highest risk group, with a global incidence rate of 85 per 100,000 person-years, reaching 108 per 100,000 person-years for patients older than 60 years. Other groups at a higher risk of SCCA include individuals who are immunosuppressed without HIV, women with a history of gynecologic cancers and precancers, women and men who have sex with women (MSW) living with HIV, and MSM without HIV [3].

Female sex and a prolonged period of immunosuppression were recognized as factors that heightened the risk of SCCA among solid organ transplant recipients; therefore, women who underwent a kidney transplant more than 10 years ago reach an incidence of approximately 50 per 100,000 person-years, surpassed only by the cohort of HIV-positive MSM [3,4].

Among women with a history of HPV-related gynecological neoplasms, those previously diagnosed with vulvar cancer are at a higher risk of SCCA compared to those with a history of vaginal or cervical cancers, with an anal cancer incidence rate of approximately 48 new cases per 100,000 per year, reflecting a similar disparity after respective precancerous lesions [3,4,5].

As expected, the incidence of SCCA among HIV-positive women and HIV-positive men who have sex with women (MSW) is higher than that among their HIV-negative counterparts but lower than that among HIV-positive MSM and is strongly age-dependent. Anal cancer risk is also consistently higher among HIV-positive MSW than among HIV-positive women, possibly due to the misclassification of male sexual preferences or practices.

Although there is considerable evidence of a close association between receptive anal intercourse and the risk of anal cancer, HIV-negative MSM represent the group for which the SCCA incidence remains the least well characterized, given that sexual practices and identity are not reported at a population level. Only a few studies have reported anal cancer incidence rates in HIV-negative MSM, with an average of 19 cases per 100,000 per year, well below that observed in many other risk groups [3].

The majority of SCCAs are attributable to carcinogenic human papillomavirus (HPV) infections, especially HPV16 and, to a lesser extent, HPV18. At the anal level, lower genotypic variability is reported than at the cervical level [6]. Similarly to cervical cancer, anal cancer progresses through detectable precursor lesions. These precursors can be identified via exfoliative sampling methods and confirmed with high-resolution anoscopy (HRA) coupled with directed biopsies [7,8].

A two-tiered classification for intraepithelial lesions has been proposed by the Lower Anogenital Squamous Terminology Standardization (LAST) Project [9]: low-grade squamous intraepithelial lesions (L-SILs) and high-grade squamous intraepithelial lesions (H-SILs). However, it has been found that anal condyloma, considered a variant of L-SILs, may also harbor foci of H-SILs or even invasive squamous cell carcinoma. The coexistence of different lesions in the same patient is due to the frequent detection of multiple HPVs, both high- and low-risk, in MSM, especially patients with HIV [10,11].

A pivotal study, the U.S. Anal Cancer HSIL Outcomes Research (ANCHOR) study, has recently shown that treating anal precancers can significantly reduce the risk of progression to anal cancer in people living with HIV (PLWH) aged 35 and older. This finding highlights the critical importance of the early detection and treatment of anal precancers to prevent invasive cancer development [12].

Despite these advancements, there are currently no nationwide guidelines for anal cancer screening. However, the New York State Department of Health AIDS Institute has updated its screening guidelines for PLWH, with the most recent update in 2022 [13]. Their recommendations specify that individuals aged 35 or older who are MSM, cisgender women, or transgender women or men should undergo annual screening. This screening should include symptom inquiry, a digital anorectal examination, and anal cytology. For cytology results showing low-grade squamous intraepithelial lesions (LSILs) or high-grade squamous intraepithelial lesions (HSILs), HRA is recommended [14,15]. In cases where cytology shows atypical squamous cells of undetermined significance (ASCUS), HPV testing should be conducted. If the test is positive for high-risk HPV (HR-HPV), or if HPV testing is unavailable, the patient should be referred for HRA [13].

Since 2017, we have begun performing primary screening for HPV-related anal cancer with a co-test that includes an anal Pap smear and HPV testing at the Sexually Transmitted Diseases (STD) Centre of the Dermatology Unit at Piero Palagi Hospital in Florence.

The aim of this study was to evaluate the correlation between HRA findings and primary screening results from co-testing in high-risk patients attending our STD outpatient clinic.

## 2. Materials and Methods

We conducted a single-center, retrospective study, collecting data from the joint multidisciplinary anal cancer clinic of Piero Palagi Hospital in Florence, between August 2019 and September 2022.

The inclusion criteria for enrolling patients in the study were MSM, regardless of their HIV status, and women with at least one risk factor for anal cancer (HIV seropositivity, a history of anal condylomas, other HPV-related conditions, or any immunosuppression status), at least 18 years old, who had already been screened at our STD outpatient clinic, with at least one of the two screening tests showing an abnormal result.

We collected a unique endoanal sample for both cytology and HPV testing using a disposable non-sterile cytobrush placed in liquid-based cytology fixative fluid (PreservCyt solution, Hologic, Marlborough, MA, USA). All samples were sent to the Regional Laboratory of Cancer Prevention of the Institute for Cancer Research, Prevention and Clinical Network (ISPRO) in Florence.

Liquid-based cytology (LBC) slides were prepared for all samples using an automated platform (ThinPrep 5000 with Autoloader, Hologic). Slides were stained with an automated platform (Leica Multistainer, Leica Biosystems Nussloch GmbH, Nußloch, Germany) using Papanicolaou staining. Anal cytological results were classified, according to the Bethesda System for Reporting Cytology (2014), as negative for intraepithelial lesions or malignancy (NILM), atypical squamous cells, of undetermined significance (ASC-US), a low-grade squamous intraepithelial lesion (LSIL), atypical squamous cells, cannot exclude a high-grade squamous intraepithelial lesion (ASC-H), atypical glandular cells (AGCs), a high-grade squamous intraepithelial lesion (HSIL), and invasive carcinoma [16].

HPV genotyping was performed on the same specimen used for anal cytology using the Anyplex II HPV 28 Detection test (Seegene, Düsseldorf, Germany). This assay is a qualitative test for the amplification, detection, and identification of 28 different HPV types, including 13 high-risk types (HPV 16, 18, 31, 33, 35, 39, 45, 51, 52, 56, 58, 59, 68), 8 low-risk types (HPV 6, 11, 40, 42, 43, 44, 54, 61), and 7 undetermined-risk types (HPV 26, 53, 66, 69, 70, 73, 82), as well as an Internal Control (IC). HRA was usually performed without a previous bowel preparation technique, with the patient in the lithotomy position; a disposable anoscope was placed into the anus with lidocaine lubrification, and then a gauze with a 5% acetic acid solution was inserted and kept for a maximum of 1 min in the anal canal while the anoscope was gently removed. After the application of the acetic acid solution, the anoscope was inserted again, and the mucosa was carefully examined with the aid of an optical colposcope (Centrel Z4) for lighting and magnification. The examination needed to be conducted quadrant by quadrant, taking care to evaluate, above all, the transition zone, smoothing the mucosa and visualizing the crypts. When anoscopy highlighted the presence of the so-called “aceto-white changes” which are characteristic of dysplastic lesions, such as flat or slightly raised or thickened areas with abnormal blood vessels, like punctuation or a mosaic vascular pattern, we applied a 3% Lugol iodine solution using a cotton swab. Regarding Lugol iodine staining, lesions considered highly suspicious of being an HSIL typically do not take up the Lugol stain and turn a yellowish color (Lugol-negative), due to their lack of glycogen. A biopsy was then performed and sent to the Pathologic Anatomy Unit for histopathological diagnosis [17].

Biopsies were classified according to the lower anogenital squamous terminology guidelines into L-SILs (anal intraepithelial neoplasia grade 1, AIN1), H-SILs (AIN2, AIN3, or carcinoma in situ), or NEG (negative) [9].

To test the statistical significance of relationships between independent categorical values, we used the χ^2^ test. To test the statistical significance of relationships between normally distributed variables, the student *t*-test for paired or unpaired values with 2 tails was used. All calculations and graphics were performed or created using Rstudio Version 1.3.959 “Middlemist Red” for Windows.

## 3. Results

During the study period, 577 HRAs were performed (divided into 454 men and 123 women). A biopsy was performed in all cases where the HRA highlighted suspicious lesions. No suspicious lesions were found in 308 patients (53.4%), for whom no biopsy was performed. The HRA results obtained with histology were as follows: 31 (5.4%) resulted in an AIN2+ diagnosis, 220 (38.1%) resulted in an AIN1 diagnosis, and 18 (3.1%) had a histological diagnosis of nonspecific inflammation. These 18 patients were also classified as “negative” and added to the 308 patients with negative HRAs without a biopsy. Overall, 326 (56.5%) turned out to be negative.

The HRA findings categorized by the anal cytological findings are reported in Table 1.

Of the 31 cases with an AIN2+ diagnosis on histological examination, 23 (71.9%) were ASCUS or worse (ASC-US+) according to the Pap test, and the remaining 8 cases (28.1%) were NILM. Of the 220 cases with an AIN1 diagnosis, 135 (61.4%) received an ASC-US+ result from the Pap test, 76 (34.5%) were NILM, and 9 (4.1%) had not had a valuable cytological examination. Of the 326 cases with a negative HRA, 56 (17.2%) received an ASC-US+ result, 253 (77.6%) had a negative cytology, and the remaining 17 (5.2%) had not had a valuable Pap test. The HRA findings categorized by the anal HPV test results are reported in Table 2.

Of the 31 cases with an AIN2+ diagnosis on histological examination, 30 (96.8%) were positive for at least one HR-HPV type, while 1 (3.2%) tested negative for both HR- and LR-HPVs. Of the 220 cases with AIN1 classifications, 150 (68.2%) were positive for at least one HR-HPV, 66 (30%) were positive only for LR-HPV types, and 4 (1.8%) were HPV-negative. Of the 326 cases with a negative HRA, 185 (56.7%) were positive for at least one HR-HPV type, 86 (26.4%) were positive only for LR-HPV types, and 55 (16.9%) were HR- and LR-HPV negative.

In our study, anal cytology alone showed a sensitivity, specificity, positive predictive value (PPV), negative predictive value (NPV), and accuracy of 74.2%, 63.3%, 10.7%, 97.6%, and 63.9%, respectively, for the detection of AIN2+ (Table 3).

- Sensitivity = 23/(23 + 8) = **74.2%.**
- Specificity = 329/(329 + 191) = **63.3%.**
- PPV = 23/(23 + 191) = **10.7%.**
- NPV = 329/(329 + 8) = **97.6%.**
- Accuracy = (23 + 329)/(23 + 329 + 191 + 8) = **63.9%.**

Anal HR-HPV testing alone showed a sensitivity of 96.8%, a specificity of 38.1%, a PPV of 8.5%, an NPV of 99.5%, and an accuracy of 41.4% for the detection of AIN2+ (Table 4).

- Sensitivity = 30/(30 + 1) = **96.8%.**
- Specificity = 198/(198 + 322) = **38.1%.**
- PPV = 30/(30 + 322) = **8.5%.**
- NPV = 198/(198 + 1) = **99.5%.**
- Accuracy = (30 + 198)/(30 + 198 + 322 + 1) = **41.4%.**

Lastly, the combination of abnormal cytology on anal PAP test with identification of infection by at least one HR-HPV strain on anal HPV test had a sensitivity of 100%, a specificity of 29.6%, a PPV of 7.8%, a NPV of 100% and an accuracy of 33.6% for detection of AIN2+ (Table 5).

- Sensitivity = 31/(31 + 0) = **100%.**
- Specificity = 154/(154 + 366) = **29.6%.**
- PPV = 31/(31 + 366) = **7.8%.**
- NPV = 154/(154 + 0) = **100%.**
- Accuracy = (31 + 154)/(31 + 154 + 366 + 0) = **33.6%.**

Focusing on the male population (*n* = 454) and their HIV status, we had two groups: 148 HIV-positive and 305 HIV-negative patients (one patient refused a HIV test). In the first one group, we had 9 AIN2+ cases and 54 AIN1 cases, while the remaining 85 HIV-positive male patients tested negative according to HRA. In the second group, we had 16 AIN2+ cases, 134 AIN1 cases, and 155 negative cases according to HRA (Table 6).

## 4. Discussion

According to the International Anal Neoplasia Society (IANS)’s consensus guidelines for anal cancer screening, anal cytology alone or with HR-HPV triage, HR-HPV testing alone or with cytology triage, and cytology and HR-HPV co-testing are all acceptable strategies for anal cancer screening [18]. Currently, there are not enough data to compare the benefits and limitations of these strategies so as to recommend a preferred option over the others.

It has always been stated that all patients reporting an abnormal anal cytology of any degree (ASC-US+) should undergo HRA, because even ASC-US or L-SIL results according to a Pap test might be associated with a histological diagnosis of AIN2+ [8,18,19,20,21].

Compared to anal cytology, cervical cytology exhibits a stronger correlation with the histopathological diagnosis, meaning that an ASCUS or LSIL classification according to a cervical Pap test hides the existence of HG-CIN in merely 5–17% of cases. In contrast, in anal cytology there is a high degree of correlation between cytological and histological findings only for HSILs, whereas low-grade lesions show weak cyto-histological agreement [21].

In our study, of the 31 cases with a histologically confirmed diagnosis of AIN2+, only 4 cases were associated with H-SILs based on the Pap test. In 15 cases, the anal Pap test showed an L-SIL, in 3 cases an ASC-US, and in one case an AGC.

What is more, in this study, the cyto-histological concordance was so poor that even in eight cases of histologically confirmed AIN2+, anal cytology produced negative results. Compared to our previous work, where we had no cases of false-negative cytology, the present study showed that cytology had lower sensitivity (74.2% vs. 100% in the previous work) and a lower PPV (10.7% vs. 15.7%) [8]. This may have been due to the larger case series in the current study or sampling issues (i.e., the cytological sample may not be representative of the lesion, especially in the case of small lesions). Usefully, adding HR-HPV testing raised the sensitivity (100%) and NPV (100%): in fact, all eight AIN2+ cases with a Pap test result of NILM were HR-HPV positive. HR-HPV testing allowed these patients to undergo HRA and not be misdiagnosed. These findings highlight the importance of PAP-HPV co-testing, even at the expense of reduced specificity and a reduced PPV [22].

It is worth noting that the two tests can be performed together, as in our case, by taking a single sample from the patient. This leads us to prefer the co-testing option, which provides more information with the convenience of a single withdrawal and a single waiting time for a response.

Even HR-HPV testing alone was more sensitive (96.8%) than cytology alone. This raises the question as to whether HR-HPV testing should play a more pivotal role in anal cancer screening akin to what is already accepted clinical practice in cervical cancer screening [23,24]. Certainly, cytology is burdened by greater inter-observer variability than HR-HPV testing, which is a fully automated real-time PCR assay [25]. From our point of view, the main limitation regarding the wide use of standalone HR-HPV testing in anal cancer screening is due to the high prevalence of anal HPV infections in the MSM population, with the subsequent risk of too many false positives in larger groups of at-risk MSM [8].

HPV vaccination programs in the female population began several years before the introduction of universal gender-neutral vaccination, enabling a shift in the screening program for HPV-related cervical cancer by switching from the Pap test to the HPV test as the primary screening method in women aged 34 to 64 years, while in younger women (under 34), cytology is still recommended. A screening program for anal cancer, organized by age groups like that for cervical cancer, is not currently feasible. In fact, while in the female population, the HPV prevalence peaks in the third decade of life and then undergoes a rapid decline with age, in the MSM population, the HPV prevalence remains high throughout adulthood, even until the sixth decade of life, when we observe the peak of the maximum incidence of SCCA [21].

This differing pattern of the HPV infection prevalence in the two populations certainly also depends on distinct sexual behavioral habits, as well as the impact of vaccination programs.

From our point of view, the use of HR-HPV testing alone as a primary screening method for anal cancer in the MSM population risks leading to many more HRAs being performed. This excessive use of clinical resources would result in increased healthcare delivery costs, making large-scale anal cancer screening programs impractical [8,21,22].

Comparing histological reports based on the HIV status only in the MSM population, which was a larger and more homogeneous group than the female one, we did not find a statistically significant difference between patients with and without HIV (Table 6). This finding was most likely due to the fact that even HIV-negative MSM included in this study had a history of high-risk sexual behaviors. Moreover, all HIV-positive MSM enrolled were on highly active antiretroviral therapy (HAART), with a good viro-immunological profile.

Anal infections by low- and high-risk HPVs were acquired at a similar rate among MSM with and without HIV, but they were both cleared more slowly by the former group. Importantly, the prolonged use of HAART (for more than 5 years) was shown to significantly reduce the risk of acquiring anal HR-HPV infections, including those caused by HPV16 and HPV18 [26,27].

According to a recent study by Kelly et al., PLWH who receive HAART seem to have a reduced prevalence of HR-HPV, and those with an undetectable HIV viral load have a decreased risk of high-grade lesion (HSIL/AIN2+) prevalence, presumably because HAART is able to lead to the spontaneous clearance of anal HPV through an earlier and more functionally complete mucosal immune reconstitution [28,29].

The promotion of early HAART initiation and strategies for maintaining adherence should be emphasized among MSM, taking into account the potential influence on their risk for HR-HPV anal infection and, ultimately, anal cancer [26,27,28,29].

This retrospective study was limited by potential data collection biases and the exclusive recruitment of patients from our STD outpatient clinic, which may have affected the generalizability of the findings. Additionally, the limited sample size may have impacted the statistical power of the results.

Longitudinal studies evaluating the cumulative risks of anal precancer and cancer are needed to understand how long a duration between negative tests provides protection. These studies are a requirement to determine evidence-based intervals for screening and the management of the screening test results.

## 5. Conclusions

This study underscores the growing significance of integrating anal cytology with HR-HPV testing for effective anal cancer screening, particularly among high-risk populations. Our results indicate that relying solely on cytology yields suboptimal sensitivity and a suboptimal PPV, limiting its effectiveness as a standalone method. Conversely, HR-HPV testing alone exhibits higher sensitivity and a higher NPV but reduced specificity and a reduced PPV. Most notably, the combination of anal cytology and HR-HPV testing—referred to as PAP-HPV co-testing—achieved 100% sensitivity and a 100% NPV, highlighting its exceptional capability for the early identification of high-grade lesions (AIN2+), at the expense of a reduced specificity and PPV.

Interestingly, the absence of notable differences in HRA outcomes between HIV-positive and HIV-negative MSM suggests that shared high-risk behaviors and the efficacy of modern HIV management may mitigate differences in the anal cancer risk across these groups.

HRA continues to serve as a critical diagnostic cornerstone in anal cancer screening protocols, providing definitive confirmation of abnormalities detected during initial screening and facilitating prompt therapeutic interventions when necessary.

In summary, our findings advocate for a customized co-testing framework that combines anal cytology and HR-HPV testing to enhance prevention and early detection efforts. This approach is especially vital for high-risk populations, where the burden of anal cancer remains a significant concern. By optimizing screening strategies, we can better identify individuals at risk and intervene early, ultimately reducing the morbidity and mortality associated with anal cancer. These results emphasize the need for tailored, evidence-based protocols to address the unique vulnerabilities of at-risk groups, ensuring that screening programs are both comprehensive and effective in clinical practice.

## Figures and Tables

**Table 1:** HRA findings categorized by anal cytological findings.

Cytology	HRA ± Histology			
	AIN2+	AIN1	NEG	Total
AGC	1	-	2	3
ASC-H	-	6	4	10
ASC-US	3	45	31	79
HSIL	4	5	-	9
LSIL	15	79	19	113
NILM	8	76	253	338
NV	-	9	17	26
Total	31	220	326	577

**Table 2:** HRA findings categorized by anal HPV test results.

HPV Status	HRA ± Histology			
	AIN2+	AIN1	NEG	Total
HR-HPV	30	150	185	365
LR-HPV	-	66	86	152
HPV-neg	1	4	55	60
Total	31	220	326	577

**Table 3:** Confusion matrix for anal Pap test detection of AIN2+.

Pap Test	HRA ± Histology	
	NEG/AIN1	AIN2+
**ASC-US+**	191	23
**NILM**	329	8

(Total = 551; 26 “not valuable” Pap tests were excluded).

**Table 4:** Confusion matrix for anal HPV test detection of AIN2+.

HPV Test	HRA ± Histology	
	NEG/AIN1	AIN2+
**HR-HPV+**	322	30
**HR-HPV−**	198	1

(Total = 551; 26 “not valuable” Pap tests were excluded).

**Table 5:** Confusion matrix for combined anal Pap test and anal HPV test detection of AIN2+.

PAP-HPV Co-Test	HRA ± Histology	
	NEG/AIN1	AIN2+
**ASC-US+ or HR-HPV+**	366	31
**NILM and HPV−**	154	0

(Total = 551; 26 “not valuable” Pap tests were excluded).

**Table 6:** HRA results based on HIV status in MSM population.

HRA ± Histology	HIV-POSITIVE (*n* = 148)	HIV-NEGATIVE (*n* = 305)	*p* Value
AIN2+	9 (6%)	16 (5.2%)	0.88
AIN1	54 (36.5%)	134 (43.9%)	0.15
NEG	85 (57.4%)	155 (50.8%)	0.22

## Data Availability

All available information is contained within the manuscript.
